# Correction: Facile fabrication of a graphene-based chemical sensor with ultrasensitivity for nitrobenzene

**DOI:** 10.1039/d4ra90041c

**Published:** 2024-04-18

**Authors:** Ali Raza, Zaka Ullah, Adnan Khalil, Rashida Batool, Sajjad Haider, Kamran Alam, Nazmina Imrose Sonil, Alvi Muhammad Rouf, Muhammad Faizan Nazar

**Affiliations:** a Department of Physics, Division of Science and Technology, University of Education Lahore 54770 Pakistan zaka.ullah@ue.edu.pk; b Institute of Physics, Khwaja Fareed University of Engineering and Information Technology Rahim Yar Khan 64200 Pakistan; c Department of Chemistry, Division of Science and Technology, University of Education Lahore 54770 Pakistan; d Chemical Engineering Department, College of Engineering, King Saud University PO Box 800 Riyadh 11421 Saudi Arabia; e Department of Chemical Engineering Materials Environment, Sapienza University of Rome Rome 00184 Italy; f State Key Laboratory of Radio Frequency Heterogeneous Integration, College of Electronics and Information Engineering, Shenzhen University Shenzhen 518060 China

## Abstract

Correction for ‘Facile fabrication of a graphene-based chemical sensor with ultrasensitivity for nitrobenzene’ by Ali Raza *et al.*, *RSC Adv.*, 2024, **14**, 9799–9804, https://doi.org/10.1039/D3RA08794H.

The authors regret that the affiliations of two authors (Alvi Muhammad Rouf and Muhammad Faizan Nazar) were incorrectly shown in the original manuscript. The corrected list of affiliations is as shown here.

The Royal Society of Chemistry apologises for these errors and any consequent inconvenience to authors and readers.

## Supplementary Material

